# Essential Attributes of Clear Aligner Therapy in terms of Appliance Configuration, Hygiene, and Pain Levels during the Pandemic: A Brief Review

**DOI:** 10.1155/2020/6677929

**Published:** 2020-12-08

**Authors:** Anand Marya, Adith Venugopal, Nikhilesh Vaid, Mohammad Khursheed Alam, Mohmed Isaqali Karobari

**Affiliations:** ^1^Department of Orthodontics, University of Puthisastra, Phnom Penh, Cambodia; ^2^European University College, DHCC, Dubai, UAE; ^3^Orthodontic Division, Preventive Dentistry Department, College of Dentistry, Jouf University, Sakaka, Saudi Arabia; ^4^Conservative Unit, School of Dental Sciences, Universiti Sains Malaysia, Health Campus, Kubang Kerian, 16150 Kelantan, Malaysia

## Abstract

Fixed orthodontic treatment has been compromised at many levels during the pandemic period, as clinics underwent a prolonged lockdown and patients could not be treated regularly. With the end of the pandemic nowhere in sight, may be it is time to put newer tools, such as clear aligner therapy, for better use. Fixed orthodontic appliances by nature are not always self-limiting, which, if left unmonitored over a long period may cause undesirable side effects, pain, and discomfort. The undesired tooth movements that may occur with arch wire-guided mechanics in addition to problems with cut wires or removed brackets may be minimized with the use of aligners. While the benefits of using aligners are for all to see, they do require extensive planning and careful evaluation of the progress. This article reviews the advantages of using aligners during the pandemic period and how it can be beneficial in helping orthodontists resume their practice.

## 1. Life and Dental Practice in the COVID-19 Era

At the beginning of this year, one could not have predicted that the entire world and life as we know it would come to a standstill. The COVID-19 pandemic has caused widespread damage to both life and property unlike any other. The aggressive nature of this virus has made containment a difficult task, and the increasing number of people getting infected by this virus is an example of the ease with which it has spread across the globe [1]. While connectivity has been the cornerstone of many businesses throughout the world, it has also been one of the main reasons this virus has been able to spread across countries with such ease [2]. While science and medicine have made a lot of advances over the past few years, lack of knowledge pertaining to the transmission of such microbes has made it extremely hard to find a cure to this problem [1, 2].

A recent study conducted at the University of Nebraska Medical Center demonstrated that this virus is transmissible via direct contact as well as by means of fomites [3]. Contact transmission may occur from inanimate object surfaces or from one individual to another or even airborne transmission if two individuals are in proximity by means of droplets. This is the reason why dentists including orthodontists are at great risk of contracting this infection during various clinical procedures such as [4]:

1. Airborne transmission during treatment
2. Indirect transmission through a contaminated instrument or surface
3. Release of aerosols from high-speed handpieces or scalers
4. Asymptomatic carriers
5. Patients may be accompanied by family or friends who could be carriers themselves

## 2. How Will Dental Clinics Be Affected?

As on the date of this correspondence, many countries have eased their lockdowns. Dental clinics have gradually started to function with inadequate or overwhelmingly confusing guidelines and advisories to ensure no spread of infection while treating patients [2, 5]. Cross infection in the clinics may lead to an exponential rise in the number of cases in the locality, thereby putting at risk lives of many [6]. Also, such incidents may lead to litigations rising from inadequate infection control measures.

## 3. Reducing Risks and Moving Forward

As far as preventive options are concerned, there are many countries which have imposed strict lockdowns, while others have enforced guidelines regarding operations during this period. Vaccines are at various stages of trials, and it would take a while before these are freely available [7]. While the virus itself is here to stay, the time has come when dental and orthodontic practices will have to resume. Carrying out procedures under risk needs considerable thought and planning. Orthodontic treatment, especially fixed orthodontic appliances by nature, is not always self-limiting, which, if left unmonitored over a long period may cause undesirable side effects. This may cause varying degrees of “round tripping” and eventual delays in treatment [8].

Studies have suggested that pain associated with the use of fixed orthodontic appliances at different stages of treatment exerted a negative influence on the quality of life of the patients [9]. The fear of pain is considered a key factor dissuading patients from seeking orthodontic treatment in the first place [10]. Soft tissue lesions and wounds caused by orthodontic appliances may be one of the factors contributing to pain [11]. In a study of 161 patients aged 12 to 17 years, Kvam et al. [12] reported that lesions caused by fixed appliances were common (76%), while severe ulcers were present among 2.5% of these patients. In this current crisis of the pandemic, with very limited follow-up adjustments, it is obvious that many of the existing orthodontic patients with fixed appliances may have broken brackets, excess wire ends, detached attachments, or fixed functional appliances, that may impinge on the soft tissue causing a lot of pain and discomfort to the patients [8].

## 4. Why Clear Aligners?

Clear aligners are an esthetic alternative to fixed braces, primarily based on the esthetic demands of patients. They seem to have found a way to maintain the comfort level of a removable appliance and maintain control over specific tooth movements by means of tooth-colored attachments that are placed over the teeth and over which the aligners fit [13]. It is also important to know that even though aligners can be taken off for a few hours, the recommended daily wear time should be at least 20 to 22 hours for maximal tooth movement [14]. Aligners are being increasingly utilized in different kinds of complex cases, and their efficiency in two-phase therapies or simple cases has been previously documented [15, 16].

Usually, orthodontic patients need to return to the clinic for their routine follow-up sessions. Unfortunately, during the current situation of the pandemic, it is not possible for the patients' to always return on their scheduled appointments. Many may even miss their follow-up visits for a couple of months or more. This may cause a lot of unwanted effects during treatment and may delay the treatment time [8, 17].

Clear aligner therapy may offer some advantages in the COVID-19 era (Table 1), but an orthodontist must also be fully aware of the limitations of aligners over tooth movement control [18]. Movements such as rotations, extrusion, and correction of large overjets have been found difficult with clear aligners [19].

It is common that a series of aligners are provided to the patient to last for a defined period before returning to the practice for evaluation and additional aligners. Some orthodontists deliver all the aligners up-front, and then they may follow treatment progress using virtual visits online or with a monitoring system [8, 25]. Even though some aligner brands may prove to be very expensive, a low-cost alternative is to use inhouse aligners that may prove more economical over the long run, as well as ensure that the patients' treatment go on as planned with predictable tooth movements. One concern with inhouse aligners, however, is that they may be less effective compared to industrially manufactured aligners.

Through this communication, we would elicit the clinical nuances and considerations that make the use of clear aligner therapy in a post-COVID era plausible, at least until a vaccine or potential cure is in sight. It may also be a good idea to consider aligners in conjunction with fixed orthodontics to correct a part of the malocclusion initially or at the end to ease finishing [15].

### 4.1. Leveling and Alignment

Leveling and alignment is done at the start of orthodontic therapy, and normally, all teeth are moved at the same time to guide them into their proper positions [8, 26]. With fixed appliances, smaller gauge NiTi wires with considerable play in the bracket slots are used, thereby increasing chances of them slipping out from the buccal tubes at the back [27]. Once these wires slip-out, there may be trauma to the soft tissues in that area or even undesired movements. This may be the prime source of soft tissue lacerations that cause discomfort or pain instantly. The use of aligners at the start of the treatment can minimize this problem, as the teeth are covered on all five surfaces: occlusal, buccal, lingual, mesial, and distal, thereby offering good control over tooth movement [14]. Also, since there are no wires used, there is very less possibility of trauma or soft tissue impingement.

### 4.2. Deep Bites

Normally, with fixed orthodontic treatment, reverse curve wires, intrusion arches etc., it may be used for correction of deep overbites. Some of the side effects of using these unchecked materials would be proclination of the anterior teeth, lingual or distal tipping of the molars etc. [28]. In case of aligners, the curve of Spee is flattened by extrusion of premolars and molars using extrusive attachments or by intruding the anteriors using intrusive attachments [13]. Since the anterior and posterior teeth are covered on all surfaces, there is a better control over anterior proclination or reciprocal lingual or distal tipping of the molars as side effects. So, the patient can continue active treatment without having to worry much about side effects of unmitigated forces [29].

### 4.3. Space Management

During the current scenario, opening or closing spaces may be a little problematic because of the use of coil springs or power chains. With fixed brackets, the use of springs or chains needs to be continuously monitored as we need a specific amount of space that needs to be opened or closed [8]. Employing aligners during this stage has the added benefit of specific individual tooth movements that is fail-safe until the next aligner change [11]. Aligners can be used very effectively to open smaller spaces, although opening bigger spaces may require additional forms of anchorage.

### 4.4. Oral Hygiene, Ligation, and Bite Blocks

While the use of stainless-steel ties for holding the arch-wires in position may be beneficial in comparison with elastic modules in terms of maintaining hygiene and securing the arch wire, with aligners, these are not needed. No brackets, no wires, no ligatures, and the flexibility to remove aligners to clean the teeth make it extremely easy and effective to maintain proper oral hygiene during this period. It is well known that fixed orthodontic appliances can alter oral microbiology, whereas clear aligner therapy has minimal effect on the growth of oral bacteria [20, 30]. Previous studies conducted to compare the microbial colonization associated with aligners and fixed orthodontic therapy have shown that there is lesser presence of microbes and reduced risk of dental caries with the use of aligners [31–33].

Similarly, aligners can be designed with bite ramps anteriorly or posteriorly without the need to place composite or prefabricated bite blocks that may cause discomfort to the patient with fixed orthodontic treatment in case the patient is unable to visit the clinic for the next few months [12]. These bite ramps can be placed on a planned number of aligners and removed in the subsequent ones to ensure proper treatment progress.

### 4.5. Extractions and Expansion

While aligners offer no alternative for extractions, when required, these must be done under strict aseptic conditions with use of high-quality personal protective equipment. Space closure can be planned out with individual tooth movements using aligners to ensure that, even during the lockdown months, the space closure is carried out effectively. In terms of expansion, aligners are a highly efficient appliance as there is no need to use additional appliances for dentoalveolar expansion [13], although skeletal expansion may need additional tooth or bone-borne appliances. Expansion in each segment is planned and carried out stagewise so that once the desired expansion is reached, the aligner becomes passive. Also, occlusal and buccal coverage of the teeth offers an additional benefit in terms of maintaining control over undesired flaring [14] during the process. The passive aligners postexpansion may also offer effective retention phase due to complete the palatal coverage.

### 4.6. Space Closure

As mentioned earlier, with aligners, space closure movements can be planned extensively to ensure that teeth are moved as desired. The reduced dependability on power chains or closed coil springs makes lesser chances for patients' turning up with emergencies such as removed brackets, broken wires, or unwanted tipping due to excessive or unmitigated forces for a longer duration, in case the patient cannot visit the clinic regularly. With aligners, however, tooth movements must be small, and lesser teeth must be moved at the same time to ensure there are minimal side effects. Most aligners offer the option to use a virtual power chain for space closure, allowing spaces to be closed, but the orthodontist needs to plan the placement of attachments carefully so that the teeth do not tip instead of undergoing bodily movement [14, 29].

### 4.7. Miniscrews

There are many fixed orthodontic cases that require the use of miniscrews for maintaining control over anchorage during retraction, protraction, or even intrusion teeth movements [8]. If proper oral hygiene is not maintained during this period, it could lead to gingival inflammation and subsequent failure of the miniscrews which is where aligners are beneficial again [7, 21, 34]. Aligners can carry many of the movements without employing the use of miniscrews. In cases that require anterior intrusion, arch distalization, or uprighting of molars etc., the use of miniscrews is unavoidable.

### 4.8. Finishing and Detailing

The number of orthodontists who employ aligners as a part of two-phase therapy is increasing because of the flexibility that comes with aligners [15, 35–37]. Aligners can be used at the beginning of the treatment, midtreatment, or even at the end to get an ideal finish. While conventional fixed orthodontics may require the use of various elastics and wire-bending to achieve a good finish, with aligners, the same can be achieved by the placement of attachments and planned individual tooth movements to ensure an ideal finish [38].

### 4.9. Delivering Torque

Most aligners can deliver positive or negative torque as desired by the treating orthodontist [35]. With fixed orthodontic treatment, it may be difficult to see the patients for the next few months and monitor the third-order changes, so using aligners with planned buccal or lingual torque depending on the stage is a good idea.

### 4.10. Interproximal Reduction

While aligners do offer a lot of benefits when it comes to interproximal reduction, the scenario is the same, but a plausible benefit is that reduction can be planned with aligners [39]. Interproximal reduction can be planned after a few aligner stages so that the patients do not have to visit the clinic physically for the next few months. With fixed orthodontics, this may be more difficult to accomplish as the orthodontist may not be able to postpone it without certain side effects.

### 4.11. Retention

After finishing a case with fixed orthodontics, normally, the brackets must be removed, and the teeth need cleaning and polishing to remove the bonding composite residue. In such a scenario, aligners again prove to be beneficial in terms of smaller attachments to clean or continued use of the last aligners that become passive once the desired movement has been achieved. Clear retainers may be fabricated using the software used for planning tooth movement, and these may be printed or formed on a cast using the treatment planning software [40]. It is always a good idea to have an additional pair of retainers made just in case the patient breaks one by accident.

### 4.12. Attachments

While fixed orthodontic treatments employ the use of brackets and wires, aligners also make use of attachments to maintain precise control over tooth movement [23]. Aligners have been utilized well in the management of Class II cases by distalization of the maxillary molars with the help of planned attachments and elastics [41]. In case any attachments are lost, a nonaerosol generating procedure may be employed to rebond the lost attachments.

### 4.13. Virtual Monitoring

With the advent of technology, a lot of virtual monitoring tools are available to orthodontists for being able to monitor patients currently undergoing treatment. With the ability to plan out the entire treatment with aligners using planning software, it may be comfortable to virtually monitor the cases and match the treatment progress with the planned tooth movement progress using the software [22, 24], in case the patient is not able to physically visit the clinic. Since fixed orthodontic movements employ a continuously adaptive treatment plan, it is difficult to gauge the progress using a virtual platform. Patients can be encouraged to take intraoral records with the help of someone at home, but the reliability and accuracy of these records taken without expertise may be questionable.

## 5. Limitations

While aligners may have several plausible benefits to offer in the current scenario, we would do well to also remember their limitations in three-dimensional tooth movement. Previous studies [24, 42] have shown that, while aligners are effective in maintaining control over intrusion of anteriors, they are less effective when it comes to extrusion. Also, they are more efficient in managing labiolingual inclination in the posterior segment compared to that in case of the anteriors. Clear aligners have also been found limited in their ability to control severe rotations especially that of rounded teeth [43].

## 6. Conclusions

No one could have foreseen the COVID-19 situation beforehand, and even now, there is no definite answer to this problem. The world as a whole and dentists as a group are in unchartered territory and to survive and come out of it better, we must adapt. Charles Darwin's theory of “Survival of the Fittest” may yet come to the fore, and as practicing orthodontists, we must employ making use of the technology available to us for our benefit as well as for minimizing the risk of cross infections. With all the plausible benefits and limitations of clear aligner therapy or fixed orthodontic treatment in mind, we must also always remember that it is not the aligners or the brackets that move teeth, but it is orthodontist with extensive training in carrying out physiological tooth movements, who have the ability and the skill to do so. We, as orthodontists, must incorporate more planning, provide better patient care, and make the most of the technology that is available today.

## Figures and Tables

**Table 1:** List of advantages of using clear aligners.

Advantages of aligners	References
(1) Aesthetically pleasing	Drake et al. [14]
(2) Better oral hygiene	Zhao et al. [20]
(3) Removable	Kravitz et al. [21]
(4) Better in terms of comfort	Zhao et al. [20]
(5) Can be used for a variety of cases	Haoili et al. [22]
(6) Invisible attachments	Garino et al. [23]
(7) Beneficial for intrusion and expansion	Tepedino et al. [24]; Haoili et al. [22]
